# Exosomes secreted from sonic hedgehog-modified bone mesenchymal stem cells facilitate the repair of rat spinal cord injuries

**DOI:** 10.1007/s00701-021-04829-9

**Published:** 2021-04-05

**Authors:** Yijia Jia, Tingsheng Lu, Qiling Chen, Xingwei Pu, Linsong Ji, Jianwen Yang, Chunshan Luo

**Affiliations:** Department of Spine Surgery, Guizhou Province Osteological Hospital, 123 Shachong South Street, Nanming District, Guiyang, 550002 Guizhou Province China

**Keywords:** Sonic hedgehog, Exosomes, Spinal cord injury, BMSCs

## Abstract

**Background:**

Spinal cord injuries (SCIs) can cause a loss of neurons and associated sensory and motor functionality below the injured site. No approaches to treating SCIs in humans have been developed to date. Exosomes are extracellular vesicles that hold promise as a potential therapeutic modality when treating such injuries. The present study was thus designed to determine whether sonic hedgehog (Shh)-overexpressing bone mesenchymal stem cell (BMSC)-derived exosomes were protective in the context of SCIs.

**Methods:**

Exosomes were extracted from control or Shh lentivirus-transduced BMSCs, yielding respective BMSC-Exo and BMSC-Shh-Exo preparations which were intravenously injected into SCI model rats. Shh expression in spinal cord tissues in these animals was then assessed via immunohistochemical staining, while Basso-Beattie-Bresnahan (BBB) scores were utilized to measure high limb motor function. Neuronal damage and regeneration within the spinal cord were additionally evaluated via terminal deoxynucleotidyl transferase dUTP nick end labeling (TUNEL), Nissl, hematoxylin and eosin, and immunofluorescent staining.

**Results:**

Both BMSC-Exo and BMSC-Shh-Exo preparations significantly increased Shh expression in the spinal cord of SCI model rats and improved BBB scores in these treated animals, while also increasing the frequencies of Nissl- and NeuN-positive neurons are reducing the numbers of apoptotic and GFAP-positive neurons. While both treatments yielded some degree of benefit to treated animals relative to untreated controls, BMSC-Shh-Exos were more beneficial than were control BMSC-Exos.

**Conclusions:**

Shh-overexpressing BMSC-derived exosomes represent an effective treatment that can facilitate SCI repair in rats.

## Introduction

Spinal cord injuries (SCIs) arrive due to severe central nervous system trauma and can result in the death of spinal cord neurons, glial scar formation, and a range of neurological symptoms including neuralgia, abnormal motor or sensory function, and high rates of disability. The mechanistic basis for SCI formation is fairly complex [10]. During the subacute phase of SCI, astrocytes become excessively activated and secrete a range of neuroinhibitory and proinflammatory factors that can drive inflammation and the apoptotic death of neurons, in turn facilitating glial scar formation and thus suppressing axonal regeneration [10, 26]. As the regenerative abilities of neurons are very limited, SCIs remain difficult to treat in clinical contexts.

Many recent advances in the use of stem cell transplantation as a treatment for SCIs have highlighted the promise of this therapeutic approach [15]. Bone marrow mesenchymal stem cells (BMSCs) are multi-potent stem cells that are capable of self-renewing and that are ideally suited to stem cell transplantation-based SCI treatment owing to their multi-regulatory effects on astrocyte activation [17, 22]. While the beneficial effects of SCI therapy were previously attributed to the homing of these stem cells to damaged tissues wherein they were able to differentiate and facilitate tissue repair, more recent evidence suggests that extracellular vesicles derived from these cells may be the primary mediators of tissue repair [14, 27]. Exosomes are 40–100 nm extracellular vesicles derived from diverse cell types that can carry specific lipids, proteins, and nucleic acids that can be delivered to recipient cells [25]. BMSC-derived exosomes can simulate many of the biological activities of their parental cells while also offering the advantages of being small, less likely to block the microvasculature, non-proliferative, and less likely to facilitate oncogenesis relative to BMSCs [1]. Furthermore, exosomes can cross the blood-brain barrier and enter circulation [20]. Lu et al. [17] demonstrated that BMSC exosomes promote recovery after spinal cord injury by improving the integrity of the blood-cord barrier. Gu et al. [6] found that BMSC-derived exosomes were able to suppress neuronal apoptotic death and facilitate functional recovery in SCI model rats. Similarly, Liu et al. [16] determined that BMSC-derived exosomes were able to suppress A1 neurotoxic reactive astrocyte activation, thereby supporting traumatic SCI repair. As such, such exosomal preparations hold great therapeutic promise for the treatment of SCI.

The sonic hedgehog (Shh) signaling pathway has been highlighted as an important regulator of neuronal regeneration following injury [29], with some data even demonstrating Shh activation as a facilitator of SCI recovery in rats [28]. Whether exosomes derived from Shh-overexpressing BMSCs can similarly promote SCI repair, however, has yet to be tested. Herein, we therefore tested the regenerative potential of such exosomal preparations in order to provide a foundation for the clinical treatment of SCI.

## Materials and methods

### Animals

In total, 40 male Sprague-Dawley rats (230–250g; Jiesijie Experimental Animal Company, Shanghai, China) were used for this study. The ethical committee of Guizhou Province Osteological Hospital (Guiyang, China) approved this study (Approval number: 2019001A).

### BMSC culture

BMSCs were isolated and cultured as in prior reports [30]. Briefly, 3-week-old rats were intraperitoneally injected with 1% pentobarbital (80 mg/kg). Euthanized rats were then disinfected using 75% ethanol, and femur and tibia bones were collected, disassociated from surrounding muscle and fascial tissues, and the bone marrow therein was harvested via rinsing with Hank’s solution. Cells were then spun don at 170×*g* for 5 min at 4 °C, resuspended in Hank’s solution, spun down again at 1050×*g* at 4 °C for 5 min, and resuspended in low-glucose DMEM supplemented with 10% FBS and 1% penicillin/streptomycin (Gibco, Grand Island, NY, USA). BMSCs were then cultured at 37 °C in a 5% CO_2_ incubator, with media being replaced every other day. BMSCs in the logarithmic phase of growth from the third passage were used for subsequent experiments.

### BMSC characterization

Flow cytometry was used to confirm the purity of BMSC preparations based upon known BMSC markers [19]. Briefly, 0.25% trypsin was used to harvest BMSCs, which were rinsed, resuspended in PBS (10^5^ cells/ml), and stained with FITC-labeled antibodies specific for CD34, CD44, CD45, or CD90 (Invitrogen, Carlsbad, CA, USA) for 30 min at 4 °C protected from light. Cells were then washed twice with PBS and assessed via flow cytometry (FACScan, BD Biosciences).

### Lentiviral transduction

Shh overexpression in BMSCs was achieved by synthesizing and cloning the Rattus norvegicus Shh(NM_017221.1) target sequences into a lentiviral vector fused to a GFP reporter sequence (Hanheng Biotechnology Company, Shanghai, China), with a control vector (Lv-vector) serving as a negative control. Lentiviral transduction was achieved by co-transfecting these plasmids into HEK293T cells along with the psPAX2 and pMD2 vectors. At 48 h post-transfection, lentivirus-containing supernatants were collected and passed through a 0.45-μm filter (Millipore, Billerica, MA, USA). BMSCs were infected with these lentiviral particles at an initial multiplicity of infection (MOI) of 25, and transduction efficiency was assessed after 48 h via western blotting.

### Exosome isolation and characterization

An Exo Quick-TC kit (SBI, USA) was used to collect BMSC-derived exosomes based upon provided directions. Transmission electron microscopy (Libra 120; Zeiss, Oberkochen, Germany) was conducted to assess exosomal ultrastructure, while western blotting was used to detect the exosomal markers CD9, CD63, and TSG101 (Abcam, UK).

### Rat SCI model development

Rats were intraperitoneally injected with 1% pentobarbital sodium (50 mg/kg), disinfected, and a 2–3 cm incision at the midline of the back was generated centered around the T10 spinous process. Surface musculature was separated to expose the underlying vertebrae, and the T10 spinal cord was fully exposed by removing the spinous processes and lamina. A striking device was then used to apply a 2 N striking force to the T10 spinal cord. The wound was then rinsed with saline solution containing penicillin and was closed. Successful SCI modeling was confirmed based upon rapid blood stasis in the spinal cord and rapid contraction and tremors in the lower limbs of model animals. Sham-operated control rats were anesthetized and had their spinal cords exposed, but no injuring force was applied.

### Groups

SCI model rats were randomized into three groups (*n* = 10/group): SCI, BMSC-Exo, and BMSC-Shh-Exo groups. At 1 h post-modeling, rats were intravenously injected with 200 μl of the appropriate exosomes (200 μg/ml) via the tail vein. Rats were administered three total injections, with injections being administered every other day. SCI model rats were instead injected with physiological saline. Rats were then injected with penicillin (2 × 10^5^ U/kg) once daily for 3 days, and urination care was provided three times per day until the resumption of active urination.

### Motor function analyses

Rat locomotor functionality following SCI modeling was assessed based upon Basso-Beattie-Bresnahan (BBB) scores [4]. Scores were independently determined by two researchers blinded to treatment group assignments and were calculated on days 1, 3, 7, 14, and 28 post-operation.

### Hematoxylin and eosin staining

Spinal cord tissue sections were fixed with 4% methyl alcohol, embedded in paraffin, sectioned, deparaffinized, and subjected to standard hematoxylin and eosin (H&E) staining. Sections were then dehydrated using ethanol and xylene, sealed using neutral resin, and assessed under an optical microscope (Olympus, Japan).

### Nissl staining

Sections were first placed into a 1:1 anhydrous ethanol:chloroform solution overnight at room temperature, after which they were sequentially placed in 100% ethanol, 95% ethanol, and distilled water. Sections were then stained for 10 min using pre-warmed tar purple (0.1%, pH = 3) at 37 °C, washed once with distilled water, differentiated for 5 min with 95% ethanol, and dehydrated in 100% anhydrous ethanol and xylene for 5 min. Sections were then mounted and examined via microscopy, with the number of motor neurons in five randomly selected fields of view of the anterior horn being counted to assess the impact of treatment on motor function.

### TUNEL staining

Terminal deoxynucleotidyl transferase dUTP nick end labeling (TUNEL) enables the visualization and quantification of cellular apoptosis by assessing the fragmentation of nuclear DNA, which occurs early during apoptosis [12]. Rat spinal cord sections (10 μm) were subjected to TUNEL staining as follows (Roche molecular Biochemicals Inc., Mannheim, Germany). Briefly, sections were treated with proteinase K and 0.3% H_2_O_2_, followed by a 1 h incubation with terminal deoxynucleotidyl transferase at 37 °C. A peroxidase-conjugated antibody was then used to probe sections for 30 min, after which a DAB substrate kit (Vector Laboratories, INC, Burlingame, CA, USA) was utilized for color development. Cells with brown nuclei were considered to be TUNEL positive, and all TUNEL-positive cells within three random 200× fields of view were counted by an observer blinded to treatment groups and expressed as a percentage of total cells.

### Immunohistochemistry

Spinal cord tissues were fixed with a 4% methyl alcohol solution, paraffin-embedded, sectioned, dewaxed, and washed with PBS. Antigen retrieval was conducted by adding samples to boiling citrate buffer (pH = 6.0) for 10 min, followed by a 15 min incubation in 3% H_2_O_2_ and blocking for 1 h with 10% BSA in PBS. Sections were then probed overnight with anti-Shh (1:500; ProteinTech, Wuhan, China) at 4 °C, followed by washing and incubation with HRP-linked goat anti-rabbit IgG for 1 h. DAB was then used for color development, and sections were counterstained using hematoxylin. A light microscope (Olympus, Tokyo, Japan) was then used to image tissue sections, and ImageJ (National Institutes of Health, Bethesda, MD, USA) was used to analyze the resultant data.

### Immunofluorescence staining

Sections of spinal cord tissue were washed three times in PBST (10 min/wash) and were then blocked for 1 h at room temperature using 5% normal goat serum. Sections were then probed with AF647 anti-NeuN (1:50; Abcam) or AF488 anti-GFAP (1:50; Abcam) rabbit antibodies overnight at room temperature. Sections were then washed thrice with PBS and probed with secondary goat anti-rabbit IgG (Invitrogen) for 1 h. Nuclei were counterstained for 2 min using DAPI, and a nonfluorescent mounting medium (Dako, Glostrup, Denmark) was then used to mount sections to slides prior to laser-scanning confocal microscopy (Olympus). A total of five random fields of view were analyzed per section to assess the numbers of NeuN- or GFAP-positive cells.

### Western blotting

Protein-containing lysates were prepared from BMSCs, spinal cord tissue sections, and exosome samples, after which protein concentrations were assessed via BCA assay kit (Beyotime Institute of Biotechnology, Jiangsu, China). Proteins were separated via 10% SDS-PAGE and transferred to nitrocellulose membranes (Millipore, Jaffrey, NH, USA), which were blocked for 1 h with 5% non-fat milk and probed overnight with antibodies specific for Shh (1:1000; ProteinTech), CD9 (1:500; Abcam), CD63 (1:500; Abcam), TSG101 (1:500; Abcam), and β-actin (1:2000; Abcam) at 4 °C. Blots were then washed, probed for 1 h with anti-rabbit IgG, and protein bands were detected with an enhanced chemiluminescence (ECL) system (Thermo Scientific, Rockford, IL, USA). β-actin was used for normalization, and ImageJ (National Institutes of Health) was used for all densitometric analyses.

### Statistical analysis

GraphPad Prism v7.0 (GraphPad, San Diego, CA, USA) was used for all statistical analyses. Data are means ± standard deviation (SD) and were compared via *t*-tests and least significant difference ANOVAs. BBB scores were compared using Mann-Whitney *U* tests. *P* < 0.05 was the significance threshold for these studies.

## Results

### Shh-overexpressing BMSC-derived exosome preparation

**Fig. 1:**
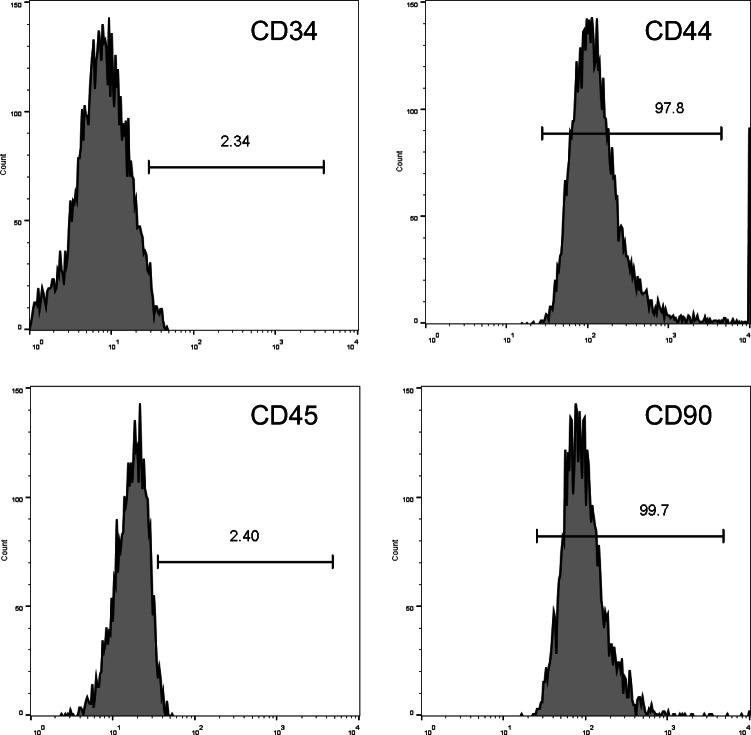
BMSC identification. The expression of the BMSC markers CD34, CD44, CD45, and CD90 on rat BMSCs was assessed via flow cytometry

**Fig. 2:**
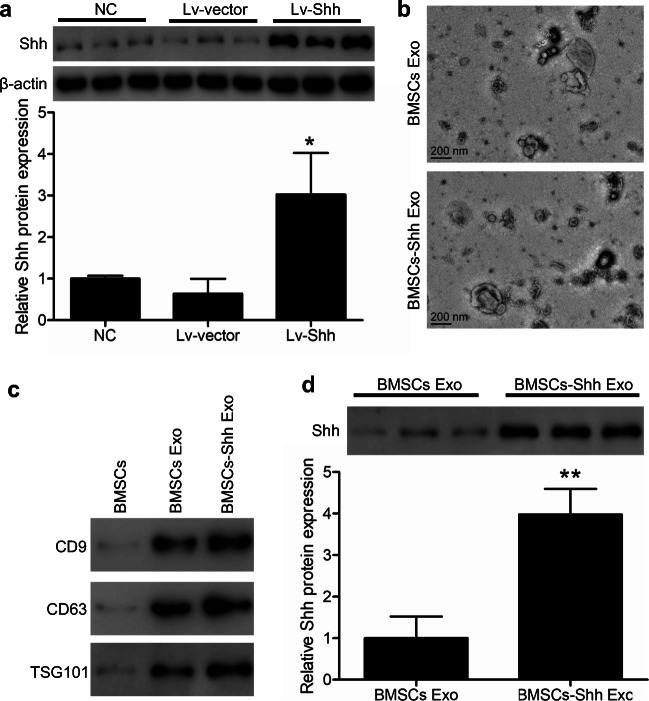
BMSC-derived exosome characterization. **a** BMSCs were transduced with lentiviruses designed to overexpress Shh or corresponding control vectors (LVvShh and Lv-vector) for 48 h, after which Shh levels were assessed via western blotting with β-actin as a loading control. *P < 0.05 vs. normal control (NC) and Lv-vector groups. **b** Exosomes derived from control or Shh-overexpressing BMSCs (BMSC-Exos and BMSC-Shh-Exos) were assessed via transmission electron microscopy. Scale bar = 200 nm. **c** Western blotting was used to assess exosomal surface markers expression (CD9, CD63, and TSG101) on BMSCs and exosomes. **d** Exosomal Shh expression was assessed via western blotting. **P < 0.01 vs. BMSC-Exos group

Following isolation and three rounds of passaging, BMSCs were collected and characterized via flow cytometry, revealing > 95% of these cells to be positive for CD44 and CD90 expression and to be negative for CD45 and CD34 expression (Fig. 1), consistent with the expected surface profiles of BMSCs. The role of Shh as a mediator of BMSC-Exo regenerative activity was then assessed by transducing BMSCs with control or Shh overexpression lentiviral vectors (Lv-vector and Lv-Shh), resulting in a significant increase in Shh expression in Lv-Shh-transduced BMSCs relative to NC and LV-vector controls (*P* < 0.05) (Fig. 2a). Exosomes derived from these cells were then characterized via transmission electron microscopy, revealing these particles to exhibit spheroid morphology (Fig. 2b). Western blotting analyses of these exosomes further confirmed that exosomes from both BMSCs and Shh-overexpressing BMSCs (BMSC-Exos and BMSC-Shh-Exos) expressed high levels of the exosomal surface proteins CD9, CD63, and TSG101 (Fig. 2c). Importantly, BMSC-Shh-Exos exhibits significantly higher Shh protein levels relative to control BMSC-Exos (*P* < 0.01) (Fig. 2d).

### BMSC-Shh-Exos facilitate the recovery of motor function in SCI model rats

**Fig. 3:**
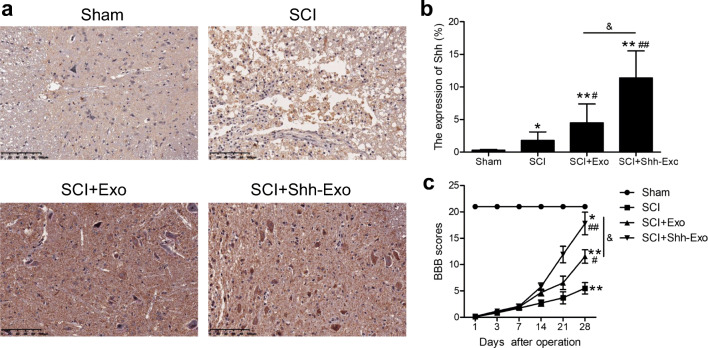
**a** Shh-positive spinal cord tissue areas in sham and SCI model rats were assessed 28 days post-operation via immunohistochemical staining. Scale bar = 100 μm. **b** Quantification of the percentage of Shh-positive area. **c** Basso-Beattie-Bresnahan (BBB) scores for rats on days 1, 3, 7, 14, 21, and 28 days post-operation. *n* = 5/group. *P < 0.05, **P < 0.01 vs. sham group. ^#^*P* < 0.05, ^##^*P* < 0.01 vs. SCI group. ^&^*P* < 0.05 vs. SCI+Exo group

To assess the ability of BMSC-Exos to protect against SCI via mediating Shh transfer, we intravenously delivered BMSC-Exos and BMSC-Shh-Exos to SCI model rats. Immunohistochemical staining was then used to assess Shh protein levels in spinal cord tissue samples from each treatment group on day 28 post-operation (Fig. 3a–b). Relative to sham controls, Shh protein levels were significantly higher in SCI model rats (*P* < 0.05) and were even first increased in rats injected with BMSC-Exos (*P* < 0.05) or BMSC-Shh-Exos (*P* < 0.01), with the latter of these treatments increasing Shh levels to the greatest extent. BBB scores were also significantly reduced in SCI model rats relative to sham controls (*P* < 0.01), while these scores were significantly increased in the BMSC-Shh-Exo group relative to the BMSC-Exo group on days 21 and 28 post-operation (*P* < 0.05) (Fig. 3c).

### Shh-overexpressing exosomes alleviate spinal cord damage in SCI model rats

**Fig. 4:**
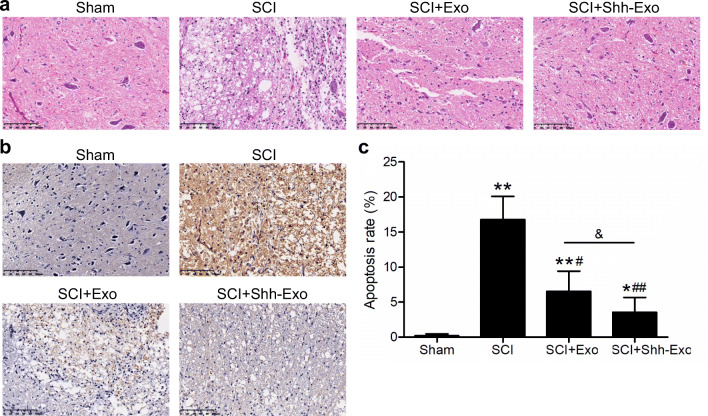
**a** Histomorphological assessment of spinal cord tissues from sham and SCI model rats on day 14 post-injection. Scale bar = 100 μm. **b** Representative images of TUNEL-stained spinal cord tissues from sham and SCI rats on day 28 post-operation. Scale bar = 100 μm. **c** Quantification of the number of TUNEL-positive cells. *n* = 5/group. *P < 0.05, **P < 0.01 vs. sham group. ^#^*P* < 0.05, ^##^*P* < 0.01 vs. SCI group. ^&^*P* < 0.05 vs. SCI+Exo group

Next, H&E staining of tissue sections from SCI model rats was performed, revealing extensive neurocyte degeneration, necrosis, and cystic cavity formation with disordered nerve fibers in the substantia alba. In contrast, samples from rats in the BMSC-Exo and BMSC-Shh-Exo groups exhibited reduced inflammation and morphologically normal spinal cords that were free of any apparent cavities. There was also evidence of nerve regeneration and complete structures in samples from rats in the BMSC-Shh-Exos group (Fig. 4a). There were also significantly more apoptotic TUNEL-positive cells in SCI model rats relative to sham controls (Fig. 4b–c), while BMSC-Exo and BMSC-Shh-Exo treatments were associated with a significant reduction in the number of TUNEL-positive cells relative to SCI model rats, with the latter of these two treatments mediating a greater reduction.

### BMSC-Shh-Exos promote neuronal regeneration

**Fig. 5:**
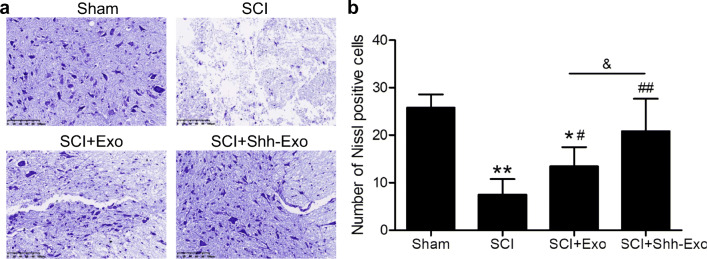
**a** Nissl staining was conducted to assess the presence of Nissl bodies in spinal cord tissues from sham and SCI model rats on day 28 post-operation. Scale bar = 100 μm. **b** Quantification of the number of Nissl-positive cells in spinal cord lesions in each group. *n* = 5/group. *P < 0.05, **P < 0.01 vs. sham group. ^#^*P* < 0.05, ^##^*P* < 0.01 vs. SCI group. ^&^*P* < 0.05 vs. SCI+Exo group

**Fig. 6:**
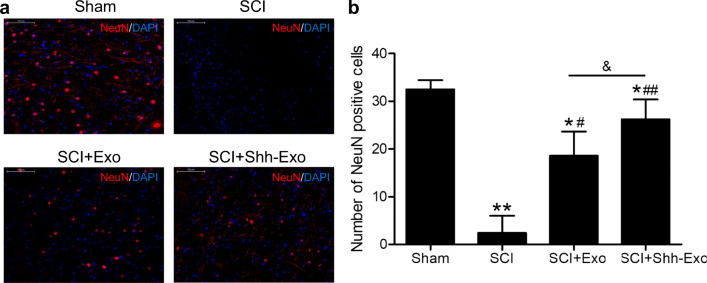
**a** NeuN labeling (red) in spinal cord tissues from sham and SCI model rats was performed on day 28 post-operation, with DAPI (blue) being used for nuclear counterstaining. Scale bar = 100 μm. **b** Quantification of the number of NeuN-positive cells in spinal cord lesions in each group. *n* = 5/group. *P < 0.05, **P < 0.01 vs. sham group. ^#^*P* < 0.05, ^##^*P* < 0.01 vs. SCI group. ^&^*P* < 0.05 vs. SCI+Exo group

**Fig. 7:**
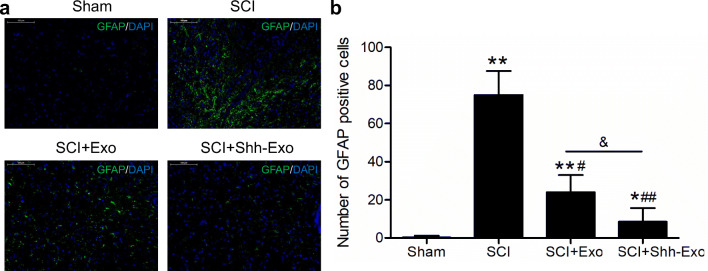
**a** GFAP labeling (green) in spinal cord tissues from sham and SCI model rats was performed on day 28 post-operation, with DAPI (blue) being used for nuclear counterstaining. Scale bar = 100 μm. **b** Quantification of the number of GFAP-positive cells in spinal cord lesions in each group. *n* = 5/group. *P < 0.05, **P < 0.01 vs. sham group. ^#^*P* < 0.05, ^##^*P* < 0.01 vs. SCI group. ^&^*P* < 0.05 vs. SCI+Exo group

To further confirm the neuroprotective benefits of BMSC-Shh-Exo treatment, Nissl staining was performed to assess the numbers of Nissl bodies in the anterior horn of the spinal cord (Fig. 5a). This analysis revealed that relative to sham controls, SCI model rats exhibited significant reductions in Nissl body numbers, whereas BMSC-Exo and BMSC-Shh-Exo treatment increased the numbers of Nissl bodies in the anterior horn of the spinal cord relative to the SCI model group, with the latter of these treatments having a more significant effect (Fig. 5b). Immunofluorescent staining also revealed low levels of NeuN expression in the spinal cord of SCI model rats (Fig. 6a), while BMSC-Exo injection significantly increased NeuN expression relative to SCI model animals on day 28 post-operation, and BMSC-Shh-Exo treatment further increased this expression relative to the BMSC-Exo group (*P* < 0.05) (Fig. 6b). Spinal cord GFAP staining exhibited opposite expression patterns to those observed for NeuN staining on day 28 post-operation (Fig. 7a–b).

## Discussion

SCIs remain a major global public health challenge, causing paralysis and serious limb dysfunction in affected individuals through primary and secondary pathological processes. Secondary injury-induced neuronal death and neuronal repair inhibition by aberrantly activated astrocytes remain the primary factors limiting effective SCI treatment [10, 21, 26]. Cell-derived exosomes represent an emerging frontier in the evaluation and treatment of SCI [3]. Cizkova et al. [2] have suggested that stem cell-derived exosomes can deliver proteins, bioactive molecules, and nucleic acids between cells, making them promising tools for SCI treatment. In a rat model of SCI, Ruppert et al. [23] found that exosomes derived from human mesenchymal stem cells altered microglial responses, reduced neuroinflammation, and improved functional recovery. Herein, we found that BMSC-Shh-Exos were able to effectively protect rats against SCI-associated pathology by promoting neuronal functional recovery, survival, and by inhibiting the excessive activation of astrocytes.

Shh is a member of the Hedgehog signaling family of proteins, which are important for organ development and embryogenesis in mammals, regulating neuronal tube directionality and limb growth [13]. Zhang et al. [28] determined that the silencing of the PTC1 and PTC2 using a lentiviral vector was sufficient to promote recovery in SCI model rats via promoting increased Shh expression and Hedgehog signaling pathway activation. We have also previously demonstrated the ability of Shh to promote neuronal and BMSC survival, to inhibit astrocyte activation, to promote axonal growth, and to bolster neurotrophic factors in BMSCs [7]. Gradilla et al. [5] determined that exosomes were able to function as vectors to transport Shh between cells, and other researchers have also found that exosomes can deliver proteins and nucleic acids to cells as a means of treating conditions such as SCI [9]. Luo et al. [18] determined that exosomes derived from G protein-coupled receptor kinase 2 interacting protein 1 (GIT1)-overexpressing BMSCs were able to suppress SCI-related glial scar formation and neuroinflammation, while also promoting axonal regeneration and suppressing apoptosis in the damaged area, indicating that the transfer of exosome-derived contents may represent an effective approach to treating SCI.

Herein, we explored the effects of exosome-facilitated Shh transfer in the treatment of SCI model SD rats. At 48 h post-transfection with an Shh-encoding lentivirus, we harvested exosomes from BMSCs and injected them into the tail vein of SCI model animals. Shh protein levels were significantly higher in the spinal cord of rats treated with BMSC-Shh-Exos relative to rats treated with control BMSC-Exos. We also found that SCI model rats exhibited increased Shh protein levels in the spinal cord relative to sham-operated controls, consistent with the findings of a prior analysis [11]. Following the administration of Shh-modified exosomes, spinal cord Shh protein levels were further increased on day 28-post SCI relative to control rats or rats administered control BMSC-Exos. BMSC-Shh-Exo treatment was also associated with significant increases in BBB scores, confirming that these exosomes improved functional recovery in SCI model rats.

The apoptotic death of neurons is commonly observed in the central nervous system following SCI and is a primary driver of the associated pathological damage [21]. How exosomal Shh affected spinal cord tissue integrity and neuronal apoptosis was assessed via H&E staining and TUNEL staining, respectively, in our SCI model rats. These analyses revealed that BMSC-Shh-Exos significantly reduced neuronal apoptosis and lesion cavity formation in the spinal cords of treated rats following SCI.

Nissl bodies can be utilized as markers of neuron functionality, while NeuN is a neuron-specific nuclear marker protein [8], and GFAP is an astrocyte activation marker that is upregulated following central nervous system trauma [8, 24]. We observed higher frequencies of Nissl bodies and NeuN-positive neurons and fewer GFAP-positive cells in spinal cord tissue samples from rats in the BMSC-Shh-Exo treatment group relative to other treatment groups, further confirming the value of these exosomal preparations as facilitators of neuronal regeneration following SCI.

## Conclusions

Overall, we found that injecting exosomes derived from Shh-overexpressing BMSCs into SCI model rats was sufficient to facilitate neuronal repair within the spinal cord of treated animals through mechanisms that may be linked to the activation of the Shh signaling pathway. Such activation may, in turn, inhibit astrocyte activation, suppress glial scar formation, promote axon and myelin sheath regeneration, and thereby improve motor function. BMSC-Shh-Exos may thus represent a viable approach to the treatment of SCIs. However, future in vitro experiments will be necessary to validate and expand upon our present findings.
